# Thoracoscopic Patch Repair of Congenital Diaphragmatic Hernia in a Neonate using Spiral Tacks: A Case Report

**Published:** 2015-07-01

**Authors:** Mario A Riquelme, Carlos D Guajardo, Marco A Juarez-Parra, Rodolfo A Elizondo, Julio C Cortinas

**Affiliations:** Hospital Christus Muguerza/UdeM, Mexico

**Keywords:** Diaphragm, Thoracoscopy, Congenital diaphragmatic hernia

## Abstract

We present a case of congenital diaphragmatic hernia that was successfully treated with spi-ral tacks using thoracoscopy. A newborn female was diagnosed with a diaphragmatic hernia at 20 weeks of gestation. The defect was surgically repaired by thoracoscopy and primary closure. On postoperative day 25, she developed respiratory distress. Chest x-ray showed a recurrence and was taken to the OR for surgical repair with spiral tacks.

## CASE REPORT

A Hispanic female, weighing 3040 g and antenatally diagnosed as right congenital diaphragmatic hernia, was born via caesarian section at 37 weeks gestation. Apgar scores were 7 and 8 at 1 and 5 minutes after birth, respectively. Few minutes after birth, she required endotracheal intubation for respiratory distress. A chest x-ray showed herniation of the liver and intestinal loops. Echocardiography showed a patent ductus arteriosus and pulmonary hypertension. After initial stabilization, a thoracoscopic repair (primarily closure with polyglactin) was performed. The patient was extubated at post-op day 8. At post-op day 25, the patient developed acute respiratory distress. Chest x-ray showed a recurrence and she was taken back to the OR. At thoracoscopy, disruption of the previously placed sutures was noticed. The recurrence was successfully repaired using a 5 x 5 cm polypropylene patch that was fixed to the costal margins and diaphragm with sutures for orientation and 12-spiral tita-nium tacks (ProTack ™ 5mm, Covidien, New Haven, CT). A chest tube was placed (Fig. 1 and 2). The postoperative recovery was uneventful. She is doing fine at three years follow-up with stable repair as shown by the position of tacks. (Fig. 3)

**Figure:**
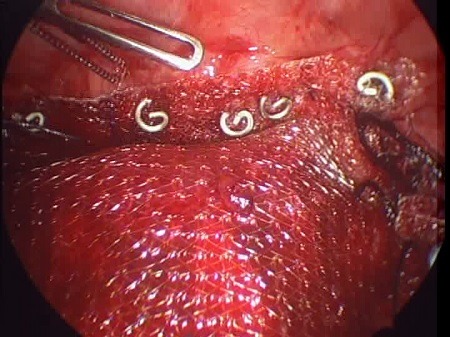
Figure 1: Thoracoscopic spiral tack application

**Figure:**
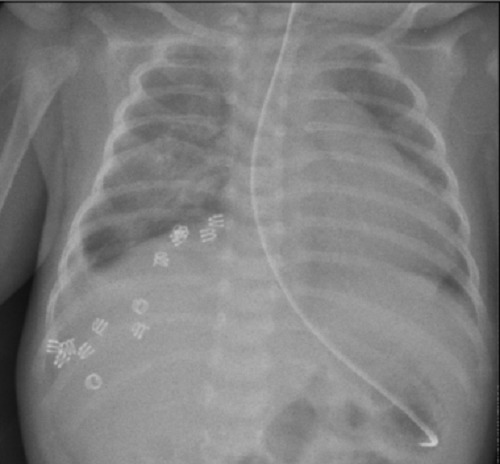
Figure 2: Chest X-ray. Post-operative spiral tack application

**Figure:**
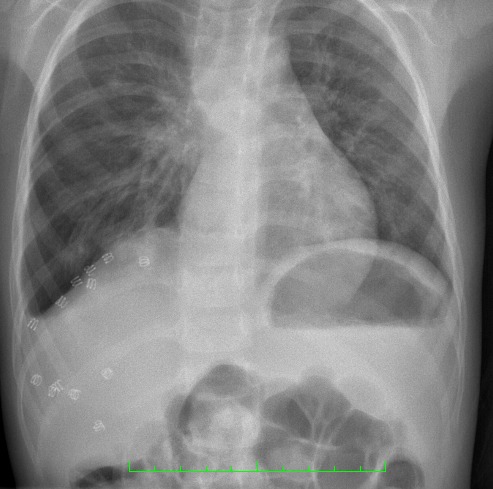
Figure 3: Chest X-ray. Three-year follow-up

## DISCUSSION

In small defects, primary closure with non-absorbable sutures is warranted, whereas for larger defects, prosthetic or muscular patches have been recommended. Regardless of the technique, the defect should be closed without tension.[1] Several materials have been pro-posed with similar results: PTFE, polypropylene, silicone, and bovine collage. Most authors recommend securing the patch to the postero-lateral aspect of the defect and fixing it to the ribcage. Recent results from a meta-analysis showed a higher recurrence rate after MIS and a subgroup of the analysis indicated higher recurrence for repairs with patch. Also, operative time was longer for MIS but postoperative mortality was higher after open surgery. [2] 

Factors that may cause recurrence include: type of patch, fixation technique, intra-abdominal pressure and excessive tension on closure, usually related to size of the defect and available adjacent tissue and prosthetic patch size. We hypothesize that the chest wall and dia-phragmatic movement influence the stability of the sutures translating into failure in primary closure of the defect. There are multiple publications on the use of tacks to prevent recur-rences and achieve better mesh fixation in ventral and inguinal hernia repair. [3]

In this case, we used a mesh and spiral tacks to repair the recurrent defect without any ad-verse effects. We recommend using tacks as an alternative to repair defects in patients with CDH. Metal tacks have the advantage of being easier to identify on a plain radiograph and monitor mesh integrity.

## Footnotes

**Source of Support:** Nil

**Conflict of Interest:** Nil

