# Thoracoscopic Patch Repair of Congenital Diaphragmatic Hernia in a Neonate Using Spiral Tacks: A Case Report

**DOI:** 10.21699/jns.v5i3.423

**Published:** 2016-07-12

**Authors:** Mario A Riquelme, Carlos D Guajardo, Marco A Juarez-Parra, Rodolfo A Elizondo, Julio C Cortinas

**Affiliations:** Hospital Christus Muguerza/UdeM, Mexico

## Erratum

It is regretted that the first and middles names for all the authors were published wrong on PUBMED for the manuscript entitled "Thoracoscopic Patch Repair of Congenital Diaphragmatic Hernia in a Neonate using Spiral Tacks: A Case Report" published as case report in Journal of Neonatal Surgery volume 4 issue 3. The correct information should be: Riquelme MA, Guajardo CD, Juarez-Parra MA, Elizondo RA, Cortinas JC.

